# Fatigue Life Estimation for Suspenders of a Three-Pylon Suspension Bridge Based on Vehicle–Bridge-Interaction Analysis

**DOI:** 10.3390/ma12162617

**Published:** 2019-08-16

**Authors:** Chuanjie Cui, Airong Chen, Rujin Ma, Benjin Wang, Shiqiao Xu

**Affiliations:** College of Civil Engineering, Tongji University, Shanghai 200092, China

**Keywords:** fatigue life, suspenders, vehicle–bridge-interaction, fatigue load spectrum, rain flow counting method, corrosion

## Abstract

Fatigue damage of suspenders is a main concern during the life-cycle maintenance of arch bridges and suspension bridges. This paper presents a practical framework for estimating the fatigue life of suspenders under repeated traffic loads by taking a three-pylon suspension bridge as an example. First, the basic theory of vehicle–bridge interaction (VBI) is introduced and a finite element model of the bridge structure is established. Second, the fatigue load spectrum is defined in detail based on the analysis of WIM (weigh-in-motion) data. And then, parametric analysis is carried out to clarify the influence of road roughness, vehicle speed, and driving lanes. Among which, the time-dependent stress laws are simulated according to the defined fatigue load spectrum and the stress range is counted through the Rain flow counting method. At last, the fatigue life of uncorroded suspenders and naturally corroded suspenders is estimated by an S–N curve and Miner cumulative damage criterion. Results reveal that the fatigue life of suspenders is more than 100 years if no corrosion happens, while less than 20 years for short suspenders considering the influence of natural corrosion.

**Highlights:**

1. Fatigue load spectrum of suspenders is defined based on the analysis of real WIM data.
2. The influence of road roughness, vehicle speed, and driving lane on suspender stress is clarified.
3. The impact of corrosion on the fatigue life of suspenders is quantified.
4. A universal program is designed for vehicle–bridge-interaction analysis of bridge structures.

## 1. Introduction

**Figure 1:**
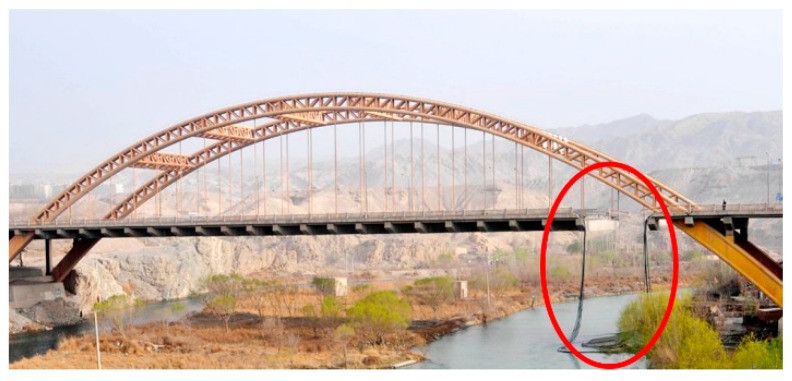
Kongque River Bridge damaged in 2011.

Suspenders are critical force transmission components for arch bridges and suspension bridges. The girder weight and live loads can be transmitted from girder to other primary force members, e.g., the main cable and arch ring, through this component [1]. However, a suspender is usually vulnerable to moving loads, e.g., pedestrians and vehicles, among which, pedestrian loads are mainly considered in footbridge design. An overview of the vibration performance of pedestrian structures is presented in [2]. While repeated traffic loads are a main cause of fractures in suspenders in highway bridges, the fracture of suspenders may induce a serious deformation of the main girder and even damage the whole structure. The Kongque River Bridge, which was damaged in 2011, is a typical accident caused by suspender fracture (Figure 1). In addition, many suspenders in arch bridges or suspension bridges have to be replaced because of different security problems. Table 1 details part of the suspender replacement projects in China [3,4]. From Table 1, corrosion of steel wires is a crucial factor resulting in the fracture of suspenders. In fact, the stress range caused by repeated traffic loads is neglectable for an intact steel wire; however, corrosion pits can lead to serious local stress concentration and then accelerate the crack nucleation and propagation rate precipitously. Pitting corrosion is one of the most common yet destructive corrosion forms of steel bridges, especially for those located in marine, industrial, and acid rain environments [5]. Thus, the influence of corrosion pits to the fatigue life of suspenders must be taken into account.

Many researches have performed experimental and theoretical investigations on the ambient vibration of highway bridges as well as model updating method to get better prediction results [6]. In terms of theoretical investigation, vehicle–bridge interaction (VBI) analysis plays an important role in traffic-induced fatigue analysis of structures. When the vehicle passes through the bridge at a certain speed, a slight vibration of the structure will be induced because of the vehicle body characteristics and road roughness, and that vibration will impact the vehicle driving condition in turn, this coupled influence between bridge and vehicles is the so-called VBI [7]. Among which, the influence of road roughness on the fatigue life of the bridge deck has been discussed in [8,9], and the statistical properties, as well as the influence of overloaded trucks, were invested by Wang L T et al. [10] and Wang W et al. [11] through VBI analysis. Alencar G et al. [12] evaluated the fatigue life of welded joints in a composite steel–concrete roadway bridge in more detail, and the results show that a detailed local stress definition is fundamental to evaluate the fatigue performance of existing roadway bridges. Li Y et al. [13] proposed an innovative time-variant dimension updating method for random traffic and carried out a case study for the dynamic analysis of bridge structures. However, almost all of the above VBI analyses aimed to clarify the fatigue life of girders instead of suspenders. Existing research on fatigue life estimation of suspenders is based on the influence line, which cannot reflect the real dynamic process taking road roughness into consideration [14]. 

Through this research, a VBI-based theory for fatigue life estimation of suspenders can be established. The structure of this paper is organized as follows. First, the VBI theory, as well as the analysis program, are introduced and illustrated briefly. Second, the finite element model of the engineering background, a three-pylon suspension bridge, is established and verified. And then, the fatigue load spectrum is defined based on the analysis of WIM data. Finally, fatigue load calculation and fatigue life estimation of suspenders with and without corrosion effects are carried out based on the above theories and definitions. 

## 2. VBI Analysis Theory

Usually, there are two approaches to simulate the VBI effect [15]: The first is to couple the VBI system and the dynamic responses can be obtained by numerical integration in the time domain, the second is to consider the vehicle and bridge separately, and their relation is contacted by the force and displacement equilibrium equations. Here the later approach is adopted. Towards this approach, the vehicle model, the bridge model, and the coupled equilibrium equation should be clarified. 

### 2.1. Vehicle Model

**Figure 2:**
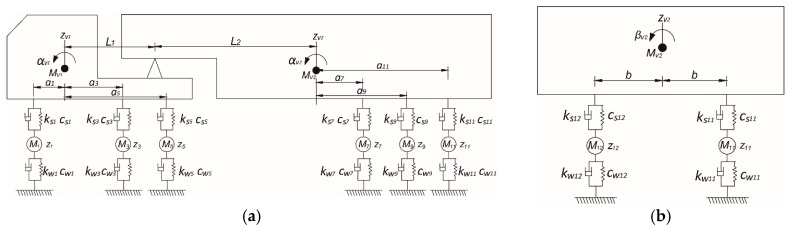
Whole vehicle model of a 6-axle trailer truck. (**a**) Length direction (**b**) Width direction.

Three kinds of vehicle models are commonly adopted in VBI analysis: the whole vehicle model, the half-vehicle model, and the single-wheel model, among which, the latter two models are both obtained by simplifying the whole vehicle model. Here the whole vehicle model was adopted to improve the analytical precision. Figure 2 plots a 6-axle trailer truck which is widely used for large cargo transportation. As can be observed, a total of 18 degrees of freedom were included in this model. The two parts of the vehicle body both have three degrees of freedom: vertical displacement, pitching rotation, and rolling rotation. In addition, each wheel contains vertical displacement freedom and two spring-damper systems.

The vibration equation of vehicle can be expressed as:

(1) $$M_{v}{\ddot{z}}_{v}+C_{v}{\dot{z}}_{v}+K_{v}z_{v}=F_{v},$$

(2) $$z_{v}=\left\{z_{v1},\alpha_{v1},\beta_{v1},z_{v2},\alpha_{v2},\beta_{v2},z_{1},z_{2},z_{3},z_{4},z_{5},z_{6},z_{7},z_{8},z_{9},z_{10},z_{11},z_{12}\right\},$$

(3) $$F_{v}=\left\{0,0,0,0,0,0,c_{w1}{\dot{z}}_{g1}+k_{w1}z_{g1},c_{w2}{\dot{z}}_{g2}+k_{w2}z_{g2},\ldots ,c_{w12}{\dot{z}}_{g12}+k_{w12}z_{g12}\right\},$$

where $M_{v},\;C_{v},\;K_{v}$ are the mass matrix, damping matrix, and stiffness matrix of vehicle, respectively; $z_{v}$ is the column vector of vehicle freedom, $z_{vj},\alpha_{vj},\beta_{vj},j=1,2$ are the vertical displacement, pitching rotation, and rolling rotation of vehicle body part j, respectively; $z_{i},\;i=1\sim 12$ is the vertical displacement of wheel i; $F_{v}$ is the column vector of external load, $k_{wi},c_{wi},i=1\sim 12$ are the stiffness coefficient and damping coefficient of wheel i, respectively; $z_{gi},\;{\dot{z}}_{gi},\;i=1\sim 12$ are the displacement excitation and velocity excitation of wheel i from the road, respectively, $z_{gi},\;{\dot{z}}_{gi}$ can be calculated by:

(4) $$z_{gi}=r\left(x_{i}\right)+z_{xi},$$

(5) $${\dot{z}}_{gi}=\frac{\partial z_{gi}}{\partial t}=\frac{\partial r\left(x_{i}\right)}{\partial t}+\frac{dz_{xi}}{dx},$$

where $r\left(x_{i}\right)$ is the road roughness of wheel i in location x, $r\left(x_{i}\right)$ will be further discussed in Section 2.4; $z_{xi}$ is the instantaneous displacement of bridge at the location of wheel i.

The two body parts in Figure 2 can be contacted by the vertical displacement compatibility equation, as shown in Equation (6). Thus, only 17 independent freedoms can be obtained for this 6-axle trailer truck.

(6) $$z_{v1}+L_{1}\alpha_{v1}=z_{v2}-L_{2}\alpha_{v2}.$$

The mass matrix, damping matrix, and stiffness matrix of the vehicle can be calculated by the principle of virtual work, the virtual work $\delta W_{I}^{v},\;\delta W_{D}^{v},\;\delta W_{E}^{v}$ done by inertial force, damping force and elastic force can be expressed by:

(7) $$\delta W_{I}^{v}=\overset{2}{\underset{j=1}{\sum }}\left(M_{vj}\;{\ddot{z}}_{vj}\delta z_{vj}+I_{vj}\;{\ddot{\alpha }}_{vj}\delta \alpha_{vj}+J_{vj}\;{\ddot{\beta }}_{vj}\delta \beta_{vj}\right)+\overset{12}{\underset{i=1}{\sum }}M_{i}\;z_{i}\delta z_{i},$$

(8) $$\delta W_{D}^{v}=\overset{12}{\underset{i=1}{\sum }}c_{si}{\dot{y}}_{i}\delta y_{i}+\overset{12}{\underset{i=1}{\sum }}c_{wi}\left({\dot{z}}_{i}-{\dot{z}}_{gi}\right)\cdot \delta \left(z_{i}-z_{gi}\right),$$

(9) $$\delta W_{E}^{v}=\overset{12}{\underset{i=1}{\sum }}k_{si}y_{i}\delta y_{i}+\overset{12}{\underset{i=1}{\sum }}k_{wi}\left(z_{i}-z_{gi}\right)\cdot \delta \left(z_{i}-z_{gi}\right),$$

where $I_{vj},\;J_{vj},\;j=1,2$ are the pitching inertia moment and rolling inertia moment of vehicle body part j, respectively; $k_{si},c_{si},i=1\sim 12$ are the stiffness coefficient and damping coefficient of suspension system i, respectively; $y_{i}$ can be calculated by Equation (10), the definition of $a_{i}$ and b can be found in Figure 2.

(10) $$y_{i}=\{\begin{array}{c} z_{v1}-a_{i}\alpha_{v1}-b\beta_{v1}-z_{i},\;i=1\sim 6\\ z_{v2}+a_{i}\alpha_{v2}-b\beta_{v2}-z_{i},\;i=7\sim 12 \end{array}.$$

### 2.2. Bridge Model

In general, large-span bridge structures can be discretely modeled by finite element methods. The main purpose of the research is to obtain time-dependent laws of suspender stress caused by vehicles. Thus, a three-dimensional beam model is acceptable to satisfy the calculation accuracy. By introducing the finite element method, the dynamic balance equation of the bridge structures can be written as:

(11) $$M_{b}{\ddot{z}}_{b}+C_{b}{\dot{z}}_{b}+K_{b}z_{b}=F_{b},$$

where $M_{b},C_{b},\;K_{b}$ are the total mass matrix, damping matrix, and stiffness matrix of the bridge structure, respectively; $C_{b}$ can be expressed as the linear combination of $M_{b}$ and $K_{b}$ according to the Rayleigh damping theory; $z_{b}$ is the column vector of bridge node; $F_{b}$ is the column vector of external force.

### 2.3. Coupled Equilibrium Equation

The vehicle model and bridge model are related through the force coupling equation and displacement coupling equation. The force coupling equation can be expressed by the vertical road force exerted from wheels, as shown in Equation (12). While the displacement coupling equation requires that the vehicle and bridge have the same vertical displacement at the wheel contact point. The instantaneous response of the bridge structure can be updated by the iterative solution method through calculating the dynamic vertical force of the wheel until the relative equation satisfies a tolerance error.

(12) $$F_{i}=c_{wi}\left({\dot{z}}_{gi}-{\dot{z}}_{i}\right)+k_{wi}\left(z_{gi}-z_{i}\right)+W_{i},$$

where $F_{i},i=1\sim 12$ is the road instantaneous force from wheel i; $W_{i},i=1\sim 12$ is the static axle load of wheel i.

### 2.4. Road Roughness

In the VBI analysis, the road roughness, which refers to the deviation degree of road surface from the reference plane, cannot be ignored. The road roughness can be assumed to be an ergodic Gaussian random process with a zero mean value, and its longitudinal distribution can be obtained by solving the power spectral density function. The power spectral density function can be expressed as Equation (13), and the solving results are presented in Equations (14)–(16).

(13) $$S\left(\omega_{k}\right)=\{\begin{array}{c} \bar{\alpha }\cdot \omega_{k}{}^{\beta },\omega_{l}<\omega_{k}<\omega_{u}\\ 0,\;\mathrm{other}\;\mathrm{values} \end{array},$$

where $S\left(\omega_{k}\right)$ is the power spectral density function of road roughness; β=1.94 is the exponential term; $\omega_{k}$ is the spatial frequency, $\omega_{l}$ and $\omega_{u}$ are the lower limit and upper limit of $\omega_{k}$; $\bar{\alpha }$ is the coefficient term which can be determined by Table 2 [16]:

(14) $$r\left(x\right)=\overset{N}{\underset{k=1}{\sum }}2\sqrt{S\left(\omega_{k}\Delta \omega \right)}\cdot \cos\left(2\pi \omega_{k}x+\varphi_{k}\right),$$

(15) $$\omega_{k}=\omega_{l}+\left(k-\frac{1}{2}\right)\Delta \omega ,\;k=1,2,\cdots \cdots ,N,$$

(16) $$\Delta \omega =\left(\omega_{u}-\omega_{l}\right)/N,$$

where $\varphi_{k}$ is a random phase angle; x is the location along the bridge; N is the number of trigonometric series.

The contact point of road surface and wheels is not only a function of location x but also the time t, which implies the velocity term of road roughness must be considered. The velocity term can be written as:

(17) $${\dot{r}}_{v}\left(x\right)=\frac{\partial r\left(x\right)}{\partial x}\cdot \frac{\partial x}{\partial t}={\dot{r}}_{x}\left(x\right)\cdot v,$$

(18) $${\dot{r}}_{x}\left(x\right)=-2\pi \omega_{k}\cdot \overset{N}{\underset{k=1}{\sum }}2\sqrt{S\left(\omega_{k}\Delta \omega \right)}\cdot \sin\left(2\pi \omega_{k}x+\varphi_{k}\right),$$

where ${\dot{r}}_{v}\left(x\right)$ is the velocity term of road roughness; ${\dot{r}}_{x}\left(x\right)$ is the change rate of road roughness; v is the vehicle speed.

### 2.5. Analysis Program and Verification

**Figure 3:**
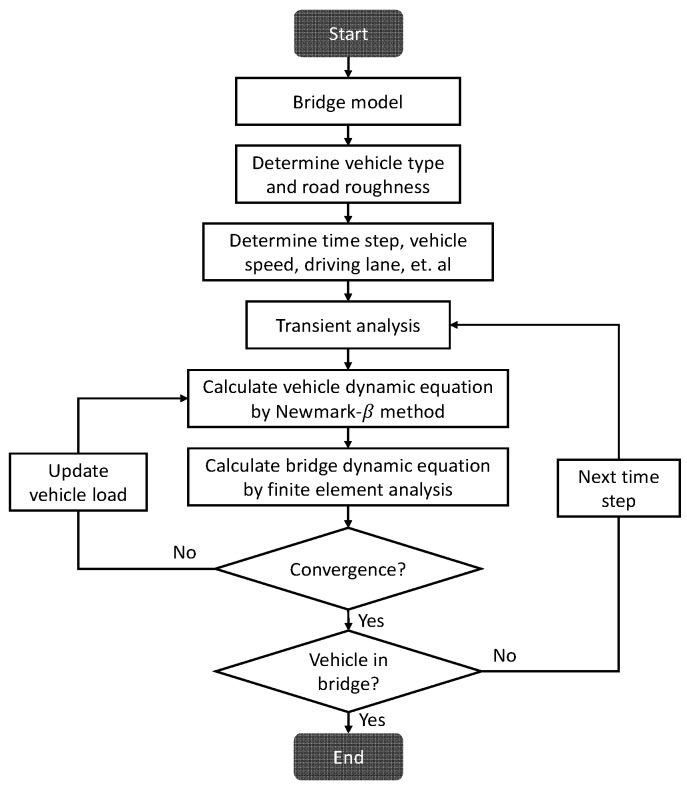
Flow chart of VBI program.

**Figure 4:**
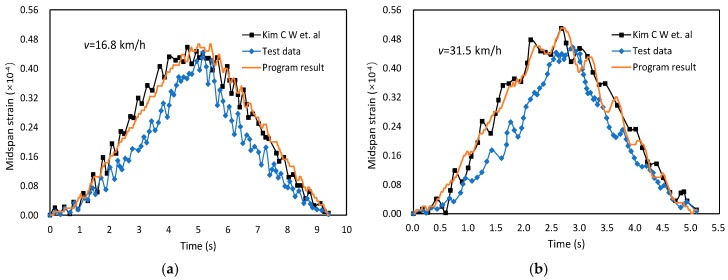
Program verification results of midspan strain. (**a**) *v* = 16.8 km/h (**b**) *v* = 31.5 km/h.

Based on the above theories, and the APDL (ANSYS Parametric Design Language)-based program for VBI analysis is established, and the flow chart of the program is presented in Figure 3. The program can be used in any bridge structure by redefining some basic relative parameters. The correction of this program is verified by a 40.4 m simply-supported bridge reported in [17]. The comparison results of midspan strain under different vehicle speeds are plotted in Figure 4. As can be observed, the calculation results in this program agree quite well with the results simulated by Kim C.W. et al. The results also agree acceptably to the test data. Thus, the program is reliable to be used for further simulation analyses.

## 3. Engineering Background and Finite Element Model

**Figure 5:**
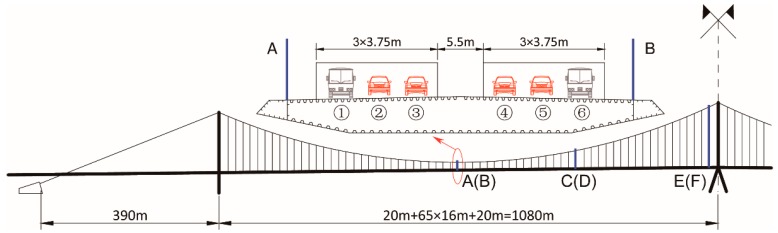
Elevation design and lane arrangement of Taizhou Yangtze River Bridge.

The Taizhou Yangtze River Bridge, with 390 m + 1080 m + 1080 m + 390 m three-pylon span arrangement, was carried out as an engineering project in this research. The bridge is located in Jiangsu Province with bi-directional and six-lane design. Figure 5 plots the elevation design and lane arrangement of the main girder. The middle pylon is constructed by steel while the side pylons are constructed by concrete. In addition, no vertical support is installed between the girder and the middle pylon. Thus, it is quite noticeable that the fatigue life of suspenders near the middle pylon is more unfavorable compared to those near the side pylons. Hence, only six suspenders, named A, B, C, D, E, and F, were used in the following VBI calculation. Among which, suspender A and B are located at the midspan, C and D are located at the quarter span, and E and F are located near the middle pylon. Suspender A, C, E are located near lane ① in the cross-section direction. 

**Figure 6:**
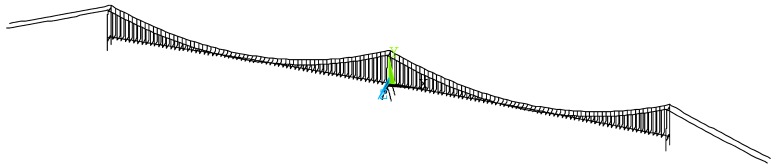
Finite element model and boundary conditions.

**Figure 7:**
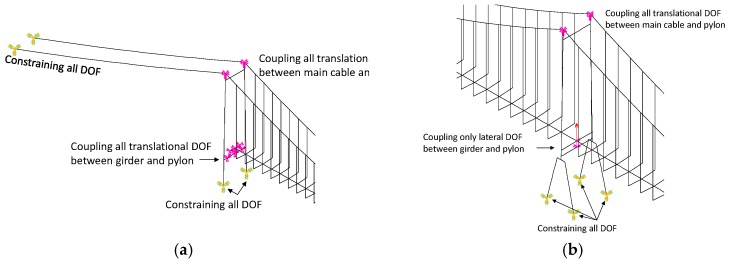
Details of boundary conditions. (**a**) near side pylon, (**b**) near middle pylon.

**Figure 8:**
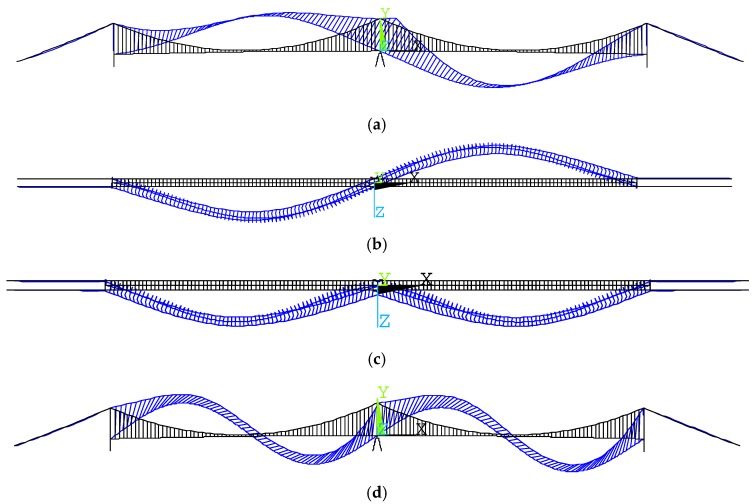
Typical mode shape simulated by the finite element model. (**a**) Order 1, elevation view, (**b**) Order 2, plan view, (**c**) Order 3, plan view, (**d**) Order 4, elevation view.

The whole bridge structure was modeled in ANSYS software, as shown in Figure 6. A total of 1857 nodes and 3027 elements were included in this model. Among which, the girder and the pylons were modeled by the BEAM4 element, and the main cable and suspenders were modeled by the LINK10 element. The vertical translation freedom, longitudinal translation freedom, and the lateral translation freedom are all coupled between the girder and the side pylons, while only the lateral translation freedom is coupled between the girder and the middle pylon. The details of boundary conditions are plotted in Figure 7. The correction of the finite element model was verified according to the field measured data. Table 3 and Figure 8 present the comparison results of vibration frequency between the finite element model and in-situ test results. It can be observed that the finite element model can well represent the actual dynamic characteristics of the bridge.

## 4. Fatigue Analysis of Suspenders

### 4.1. Definition of Fatigue Load Spectrum

**Figure 9:**
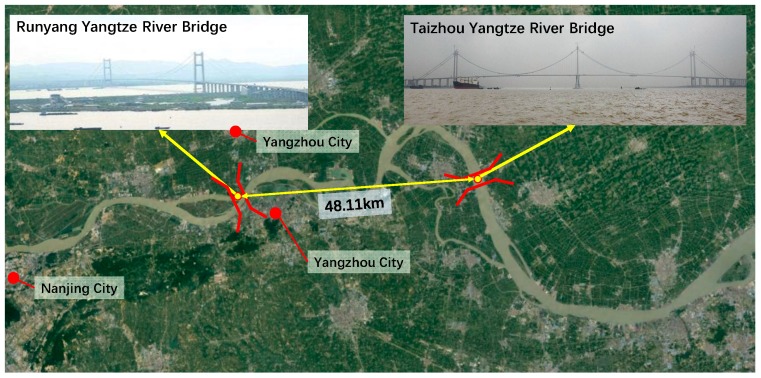
Location of Runyang Bridge and Taizhou Yangtze River Bridge.

The time-dependent stress of suspenders induced by repeated traffic loads can be influenced by many complicated factors, e.g., axle load, the number of axles, the wheelbase, vehicle speed, and the driving lane. The above vehicle information can be well recorded by WIM technology. However, no WIM system was installed in the analyzed engineering project, Taizhou Yangtze River Bridge. Here the WIM system in Runyang Bridge was adopted as the vehicle load data approximately. The geographic location of these two bridges is very close, as shown in Figure 9. Moreover, they both cross the Yangtze River and undertake similar transportation functions. Hence, the WIM data in Runyang Bridge can well reflect the traffic flow in this project. Considering the vehicle type in a real bridge and the undetermined parameters for each vehicle are rather enormous, it is almost impossible to consider all vehicle types accurately. Here the fatigue load spectrum was determined at first by selecting typical vehicle types based on the analysis of the WIM data. 

**Figure 10:**
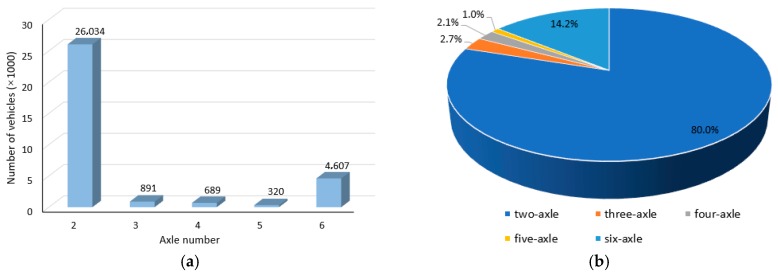
Axle distribution on 16 March 2016. (**a**) Histogram (**b**) Sector diagram.

Here six typical recording dates, 16 March 2016 (Wednesday), 11 November 2016 (Thursday), 14 May 2016 (Saturday), 6 November 2016 (Sunday), 1 May 2016 (International Labor Day), 1 October 2016 (China National Day), are selected for traffic flow analysis. The recording data distinguished by axle number is detailed in Table 4. And taking 16 March 2016 (Wednesday) as an example, Figure 10 shows the histogram and sector diagram of axle distribution. It can be observed from Table 2 that the total number of vehicles on legal holidays was much larger than that of the ordinary days. With regard to the proportion of different axle number, the proportion of two-axle vehicle on legal holidays was slightly higher compared to ordinary days, which is quite reasonable since people tend to return to their hometown or travel by driving cars at those holidays. Considering such holidays are not common, the fatigue load spectrum should be determined based on the data of ordinary days. According to the proportions counted in Table 4, the proportion of three-axle, four-axle, and five-axle vehicles can be neglected, and the traffic flow can be regarded as 80% 2-axle vehicles and 20% 6-axle vehicles approximately. 

**Figure 11:**
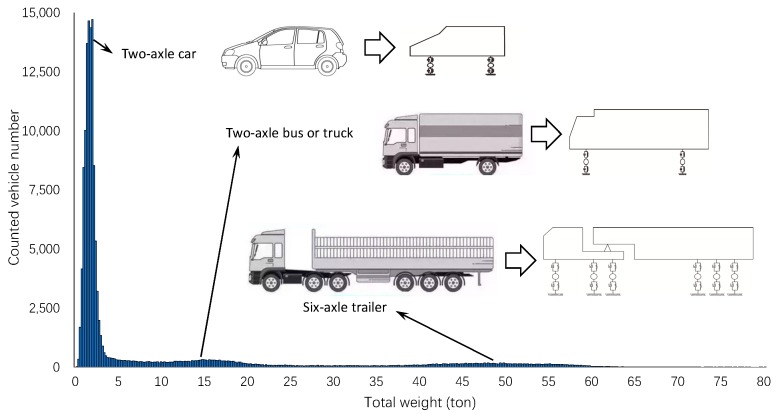
Vehicle weight distribution of ordinary days.

A further statistic on the distribution of vehicle weight of the above four ordinary days is presented in Figure 11. As can be observed, the vehicle weight had three obvious peak values: the first peak was concentrated from 0.5 tons to 3 tons, representing the traffic flow of household cars; the second peak was concentrated from 10 tons to 20 tons, representing the traffic flow of buses and container trucks; and the third peak was concentrated from 40 tons to 60 tons, representing the traffic flow of heavy-duty trailers. To simplify the VBI analysis, a 1.5 tons car (denoted by car_1.5) was selected to represent the first peak, a 15 tons truck (denoted by truck_15) was selected to represent the second peak, and a 50 tons trailer (denoted by trailer_50) was selected to represent the third peak. It can be determined that car_1.5:truck_15:trailer_50 ratio equals 0.65:0.15:0.20, approximately.

**Figure 12:**
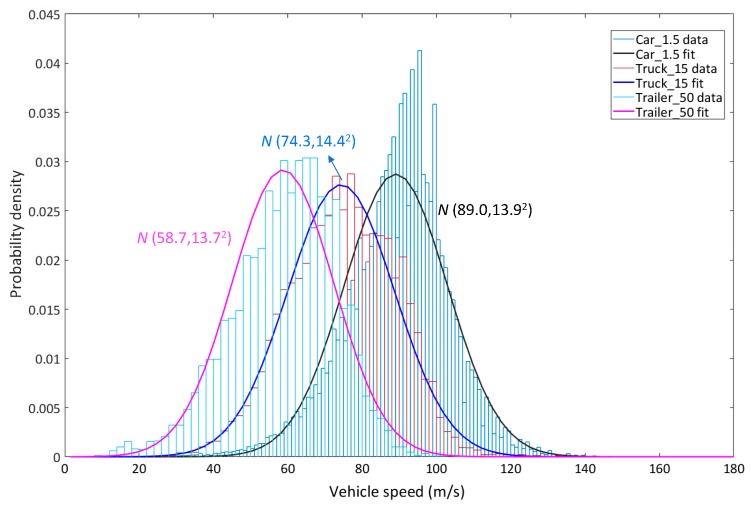
Distribution form of vehicle speed of the three typical vehicle types.

**Figure 13:**
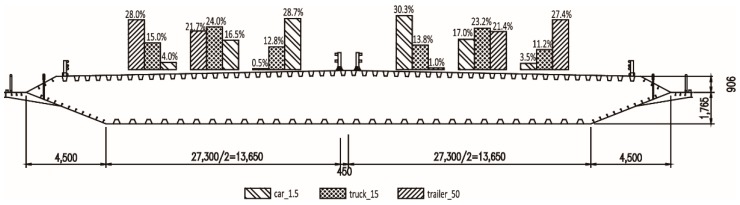
Distribution proportion of the three typical vehicle types.

Figure 12 presents the statistical results of vehicle speed of the above three typical vehicle types. As can be observed, the vehicle speed fits quite well with Normal distribution. The relative parameters of Normal distribution were calculated and presented in Table 5. Figure 13 presents the lane distribution of the above three vehicle types. Results show that the car_1.5 was mainly concentrated on the innermost two lanes while the trailer_50 was mainly concentrated on the outermost two lanes. The distribution proportion is in accordance with the design purpose of different lanes.

Thus, the probability-based fatigue load spectrum can be defined according to the counted results in Figure 11, Figure 12 and Figure 13.

### 4.2. Definition of Vehicle Parameters

The essential vehicle parameters in VBI analysis can be founded in [17,18,19,20,21,22] and summarized in Table 6.

### 4.3. Parametric Analysis

Based on the VBI program defined in Section 2.5, the finite element model established in Section 3, and the fatigue load spectrum defined in Section 4.1, the fatigue load of each suspender can be calculated straightforwardly. At first, a parametric analysis was carried out to clarify the influence of road roughness, vehicle speed, and driving lane. For parametric analysis, the trailer_50 was adopted for driving from one side to another of the bridge. 

#### 4.3.1. Influence of Road Roughness

**Figure 14:**
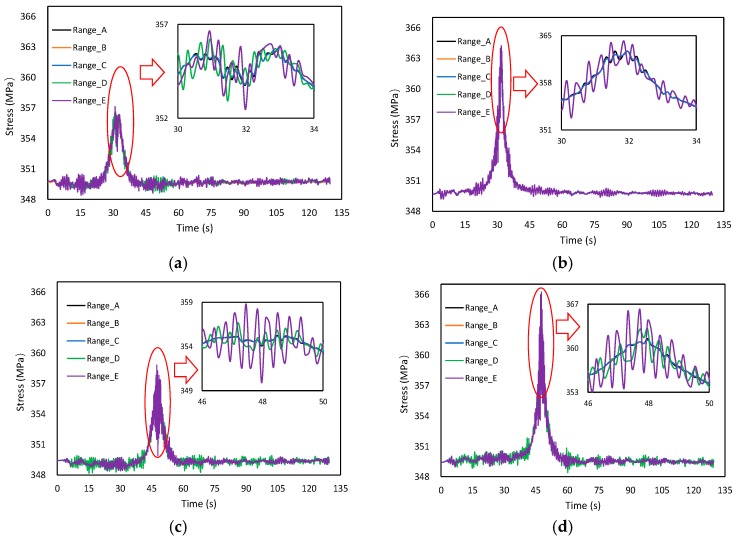

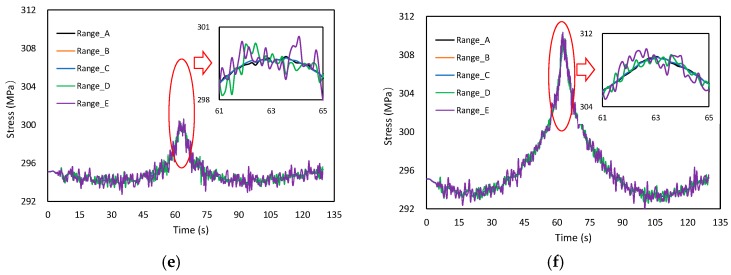
Time-dependent laws of suspender stress with different road roughness. (**a**) Suspender A, (**b**) Suspender B, (**c**) Suspender C, (**d**) Suspender D, (**e**) Suspender E, (**f**) Suspender F.

Assuming that the trailer_50 runs with a constant speed on lane ① from one side to another, and the vehicle speed is 60 km/h, Figure 14 shows the time-dependent laws of suspender stress with different road roughness levels. In general, a noticeable peak value can be observed when the trailer passes through the suspender. The overall stress range for suspenders away from the driving lane (Suspender B, D, F) was higher than that of near the driving lane (Suspender A, C, E). The road roughness had an obvious effect on the local stress range. With an increase in road roughness, the local stress range of suspenders also increased. The suspenders at the quarter span were most sensitive to the change of road roughness compared to the suspenders near the middle pylon. 

#### 4.3.2. Influence of Vehicle Speed

**Figure 15:**
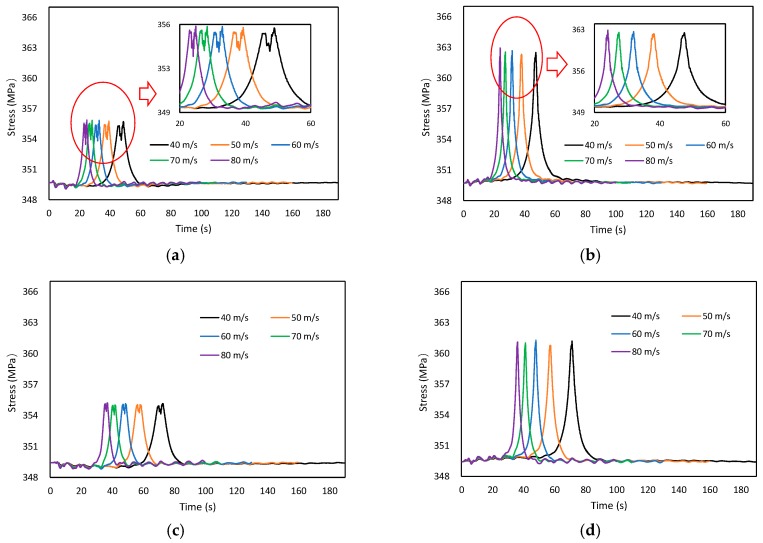

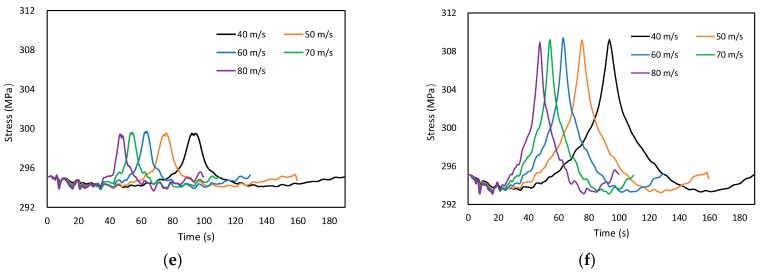
Time-dependent laws of suspender stress with different vehicle speeds. (**a**) Suspender A, (**b**) Suspender B, (**c**) Suspender C, (**d**) Suspender D, (**e**) Suspender E, (**f**) Suspender F.

Assuming that the trailer_50 runs with different constant speeds on lane ① from one side to another, and the road roughness is Range_C, Figure 15 shows the time-dependent laws of suspender stress with different vehicle speeds. As can be observed, the vehicle speed had limited influence on stress range, which almost can be ignored in the VBI analysis.

#### 4.3.3. Influence of Driving Lane

**Figure 16:**
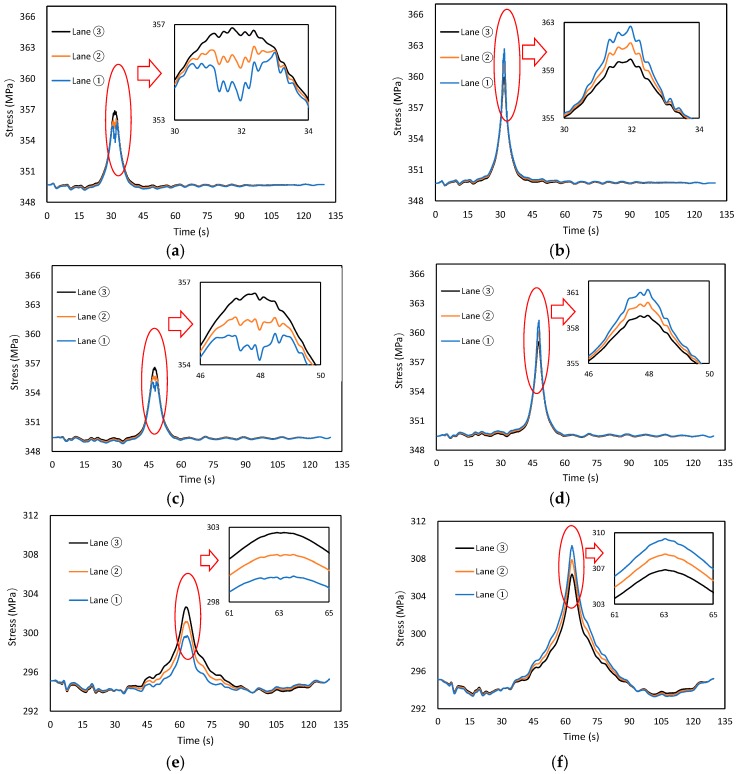
Time-dependent laws of suspender stress in different driving lanes. (**a**) Suspender A, (**b**) Suspender B, (**c**) Suspender C, (**d**) Suspender D, (**e**) Suspender E, (**f**) Suspender F.

Assuming that the trailer_50 runs with a constant speed from one side to another, the vehicle speed is 60 km/h, and the road roughness is Range_C, Figure 16 shows the time-dependent laws of suspender stress in different driving lanes. As can be observed, for the suspenders away from the driving lane, the overall stress range decreased with the decrease of eccentricity, while opposite trends can be observed for the suspenders near the driving lane. 

### 4.4. Fatigue Load Calculation

Assuming that the total daily traffic volume in bridge follows the Poisson process with a parameter λ, the WIM data in July 2016 is adopted to estimate the parameter λ considering no legal holiday in the month. It can be calculated that λ=34,390 per day. According to the fatigue load spectrum defined in Section 4.1, the traffic flow per second for these three vehicle types was $\lambda_{\mathrm{car}\_1.5}=0.26,\lambda_{\mathrm{truck}\_15}=0.06,\;\lambda_{\mathrm{trailer}\_50}=0.08$. Then, the entering time for each vehicle can be simulated by the Poisson process and the simulation program was designed in MATLAB. Similarly, the speed and driving lane for each vehicle can also be simulated based on the definition in Figure 12 and Figure 13. 

**Figure 17:**
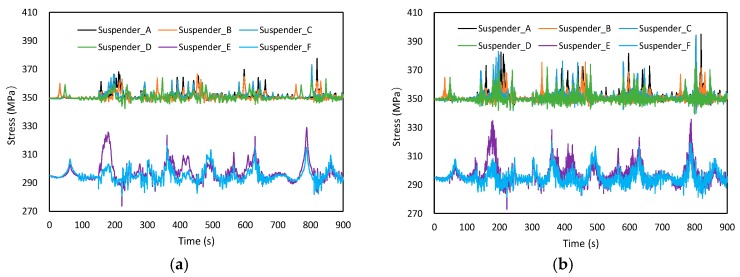
Time-dependent laws of suspender stress under defined fatigue load spectrum. (**a**) Range_A, (**b**) Range_E.

**Figure 18:**
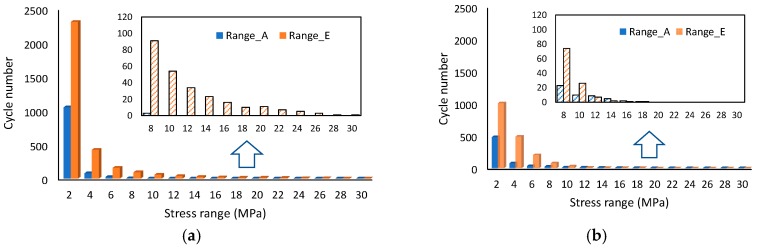
Counting results of different stress range by the Rain flow counting method. (**a**) Suspender A, (**b**) Suspender E.

Two different road roughness levels, Range_A and Range_E, were selected in VBI analysis, and the simulation time was chosen to be 15 min. Figure 17 presents the time-dependent laws of suspender stress based on the defined fatigue load spectrum. Similar to the parametric analysis results, the stress range of different suspenders also increased when the road roughness changed to be Range_E. The quantitative influence on fatigue life can be evaluated through the Rain flow counting method [23,24]. The program of the Rain flow counting method was designed in MATLAB, and the counting results are presented in Figure 18 by using Suspender A and E as the example. In addition, compared to the results of Range_A, higher stress ranges can be observed in Range_E.

### 4.5. Fatigue Life Estimation

Based on the statistical results of stress range counted by the Rain flow counting method, the Miner cumulative damage criterion can be introduced to estimate the fatigue life of suspenders under non-corrosion damage conditions.

(19) $$D^{\prime }=\sum \frac{n_{i}}{N_{i}},$$

where D′ is the damage degree; $D^{\prime }=1$ when the component is totally damaged; $n_{i}$ is the real loading times; $N_{i}$ is the ultimate loading which can be determined by the S–N curve; subscript i is the relative stress range.

#### 4.5.1. Fatigue Life without Corrosion Effect

The suspenders in this bridge are composed by high tensile parallel steel wires with a diameter of 5.25 mm and yield strength of 1670 MPa. To simplify the analysis, the following two assumptions were adopted:

(1) The S–N curve of high-strength steel wires can be directly used for fatigue life estimation of suspenders. This assumption is acceptable, considering no stranding treatment is contained in the parallel steel wires.
(2) Once the most unfavorable steel wire is broken, the relative suspender is regarded as fractured. In other words, the fatigue life of suspenders is equal to the most unfavorable inner steel wire.

The experiment on high-strength steel wires with different heat treatment methods and different surface treatment conditions carried out by Stephen S.M. et al. [25] showed that the stress range dominates the fatigue life of steel wires and the influence of mean stress is neglectable if the mean stress is less than 1380 MPa. Hence, the S–N curve can be concluded in Equation (20) based on the fatigue test carried out by Zhang J.N. [26] with the same specification of steel wires. The fatigue life estimation results of different suspenders are presented in Table 7. It can be observed that the fatigue life of suspenders under the condition of Range_A was much larger than those of Range_E. The suspenders at the midspan and quarter span were quite sensitive to road roughness while the suspenders near middle pylon changed slightly with different road roughness, that is in accordance with the simulation results in Figure 14, where more severe local vibration can be observed with higher road roughness for suspender A–D. Table 7 shows that even under the most unfavorable condition, the fatigue life of suspenders is more than 100 years.

(20) lg N=12.66−2.97 lg S

**Figure 19:**
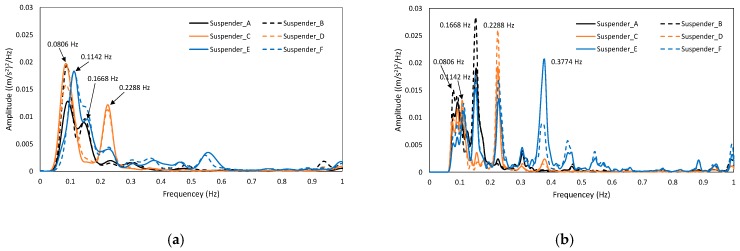
Spectrum analysis results of different road roughness. (**a**) Range_A, (**b**) Range_E.

Figure 19 plots the spectrum analysis results of the simulated time-dependent laws in Figure 18. As can be observed, when the road roughness changed from Range_A to Range_E, the vibration of suspenders tended to be controlled by the high-order mode shapes of the structure, and this trend was more obvious in the vicinity of the middle pylon. The participation factor of the high-order mode shape is generally neglectable in a real structure. Thus, it is reasonable that Suspender E and F have lower sensitivity to the change of the road roughness, as calculated in Table 7.

#### 4.5.2. Fatigue Life Considering Corrosion Effects

The suspenders have never been replaced since opening to traffic in 2012. Therefore, it is nearly impossible to quantitatively test the remaining fatigue life of those suspenders. Zheng X.L. [27] carried out a fatigue performance test on high-strength steel wires with the same specification from a three-span arch bridge opened to traffic for 13 years. The suspenders were seriously corroded because of the water seepage in the upper anchor. The observation results by scanning electron microscope indicate that the maximum pitting depth had already exceeded 300 μm. The tested S–N curve of those steel wires is:

(21) lg N=8.388−0.578 lg S,

The recalculation results of fatigue life based on Equation (21) are presented in Table 8. It can be observed that even under the condition of Range_A, the remaining fatigue life is less than five years. Considering the bridge had already been open to traffic for 13 years, the total fatigue life can be estimated to be 15 to 20 years. That fatigue life is in accordance with many real suspender replacement projects as listed in Table 1. The comparison results between Table 7 and Table 8 indicate that corrosion has a significant influence on the fatigue life of suspenders, especially for those in the vicinity of the midspan, in other words, the short suspenders. Another noticeable phenomenon is that in Range_A, the fatigue life of suspender A–D is much larger than suspender E–F without corrosion effects, while suspender A–D becomes more unfavorable if considering the influence of corrosion. The reason is that in the condition of Range_A, a higher stress range can be observed for suspender E–F, while the total number of different stress ranges is far less than that of suspender A–D, as presented in Figure 18. Furthermore, it can be observed that the slope of lg N−lg S curve in Equation (21) is much lower compared to Equation (20), which indicates that the influence of lower stress range tends to be more obvious when considering the corrosion effects. That leads to the remaining fatigue life of suspender A–D being much shorter than that of suspender E–F with the growth of corrosion pits.

In conclusion, Table 7 and Table 8 show that the seepage and corrosion conditions must be carefully detected and recorded in daily inspection, and replacement measures must be carried out for the seriously corroded suspenders.

## 5. Conclusions and Discussion

In this research, the fatigue life for suspenders of a three-pylon suspension bridge was estimated based on VBI analysis, and the following conclusions can be drawn:

(1) Through parametric analysis, the road roughness has an obvious effect on the local stress range especially for the suspenders at midspan and quarter span, while the vehicle speed has limited influence on the stress range in this three-pylon suspension bridge. In terms of the influence of driving lane, for the suspenders away from the driving lane, the overall stress range decreases with the decrease of eccentricity, while opposite trends can be observed for those near the driving lane.
(2) The simulation results of time-dependent suspender stress under a defined fatigue load spectrum show that compared to the results of Range_A, higher stress ranges can be observed in Range_E. For the condition of Range_A, the maximum stress range is no more than 15 MPa, while for Range_E, the maximum stress range exceeds 30 MPa.
(3) The fatigue life estimation results show that corrosion can strongly reduce the fatigue life of suspenders. For the uncorroded suspenders, the fatigue life is more than 100 years even under the most unfavorable conditions, while the fatigue life is less than 20 years if considering natural corrosion effects.

This paper outlines a practical framework for estimating the fatigue life of suspenders by VBI analysis. However, some details in the research still need to be further studied. Future research will concentrate on the detailed verification and promotional possibility of the defined fatigue load spectrum. In addition, the corrosion evolution laws and the coupled effects of corrosion and fatigue should be further discussed. 

## Figures and Tables

**Table 1:** Some famous suspender replacement projects in China.

Number	Bridge	Reason for Suspender Replacement	Operation Time (year)
1	Yellow River Highway Bridge in Jinan, Shandong Province	Serious corrosion of steel wires	13
2	Haiyin Bridge in Guangzhou, Guangdong Province	Suspender fracture	7
3	Yongjiang Bridge in Ningbo, Zhejiang Province	Suspender force exceeds the limit value	17
4	Yunlong Bridge in Wuzhou, Guangxi Province	Sheath damage and wire corrosion	11
5	Jiangyin Yangtze River Bridge, Jiangsu Province	Sheath damage and wire corrosion	10

**Table 2:** Definition of coefficient term $\bar{\alpha }$.

Standards of Road Roughness	$\bar{\alpha }\;\left(m^{2}/\left(m/\mathrm{cycle}\right)\right)$
Very good (Range A)	$\leq 0.24\times 10^{-6}$
Good (Range B)	$\left(0.24\times 10^{-6},\;1.0\times 10^{-6}\right]$
Average (Range C)	$\left(1.0\times 10^{-6},\;4.0\times 10^{-6}\right]$
Poor (Range D)	$\left(4.0\times 10^{-6},\;16.0\times 10^{-6}\right]$
Very poor (Range E)	$>16.0\times 10^{-6}$

**Table 3:** Comparison results of vibration frequency.

Order	Mode Shape	Test Frequency (Hz)	Calculation Frequency (Hz)	Error
1	1st Antisymmetric vertical vibration of the girder	0.0808	0.0806	0.25%
2	1st Antisymmetric lateral vibration of the girder	0.0915	0.0887	3.06%
3	1st Symmetric lateral vibration of the girder	0.1053	0.1119	−6.27%
4	2nd Antisymmetric vertical vibration of the girder	0.1190	0.1142	4.03%
5	1st Symmetric vertical vibration of the girder	0.1202	0.1160	3.49%

**Table 4:** Statistical results of axle number distribution.

Date	Total Number of Vehicles	Proportion of Different Axle Number				
		Two-Axle	Three-Axle	Four-Axle	Five-Axle	Six-Axle
16 May 2016 (Wednesday)	32,541	80.0%	2.7%	2.1%	1.0%	14.2%
11 August 2016 (Thursday)	35,866	80.7%	2.8%	2.1%	0.8%	13.6%
14 May 2016 (Saturday)	36,914	83.3%	2.0%	1.6%	0.6%	12.5%
6 November 2016 (Sunday)	37,692	82.1%	2.2%	1.7%	0.4%	13.6%
1 May 2016 (International Labor Day)	79,952	94.6%	0.6%	0.6%	0.1%	4.1%
1 October 2016 (China National Day)	87,489	93.6%	0.8%	0.7%	0.1%	4.8%

**Table 5:** Distribution form and relative parameters of vehicle speed.

Vehicle Type	Distribution Form of Speed	Mean Value μ	Standard Deviation σ
car_1.5	Normal distribution	89.0	13.9
truck_15	Normal distribution	74.3	14.4
trailer_50	Normal distribution	58.7	13.7

**Table 6:** Parameters of vehicles (the meaning of symbols can be observed in Figure 2).

Parameter	Symbol	Unit	Vehicle Type		
			car_1.5	truck_15	trailer_50
**Total Weight**	G	ton	1.5	15	50
Distance between front axle and gravity center of body ①	$a_{1},\;a_{2}$	m	1.25	2.59	1.84
Distance between middle axle ① and gravity center of body ①	$a_{3},\;a_{4}$	m	-	-	2.23
Distance between middle axle ② and gravity center of body ①	$a_{5},\;a_{6}$	m	-	-	3.55
Distance between rear axle ① and gravity center of body ①	$a_{7},\;a_{8}$	m	1.51	1.41	-
Distance between rear axle ① and gravity center of body ②	$a_{7},\;a_{8}$	m	-	-	2.82
Distance between rear axle ② and gravity center of body ②	$a_{9},\;a_{10}$	m	-	-	4.06
Distance between rear axle ③ and gravity center of body ②	$a_{11},\;a_{12}$	m	-	-	5.30
Distance between connection point and gravity center of body ①	$L_{1}$	m	-	-	2.89
Distance between connection point and gravity center of body ②	$L_{2}$	m	-	-	6.53
Tread	2b	m	1.48	1.80	4.74
Mass of vehicle body ①	$M_{v1}$	ton	1.33	12	6.45
Pitching moment of inertia of vehicle body ①	$I_{v1}$	ton·m^2^	2.44	17.3	0
Rolling moment of inertia of vehicle body ①	$J_{v1}$	ton·m^2^	0.38	5.7	0
Mass of vehicle body ②	$M_{v2}$	ton	-	-	32.0
Pitching moment of inertia of vehicle body ②	$I_{v2}$	ton·m^2^	-	-	40
Rolling moment of inertia of vehicle body ②	$J_{v2}$	ton·m^2^	-	-	8.2
Un-sprung mass of front axle	$M_{1},M_{2}$	ton	0.04	0.5	0.525
Un-sprung mass of middle axle	$M_{3}\sim M_{6}$	ton	-	-	1.05
Un-sprung mass of rear axle	$M_{7}\sim M_{12}$	ton	0.045	1.0	1.05
Spring constant of suspension of front axle	$k_{s1},k_{s2}$	kN/m	17	250	250
Spring constant of suspension of middle axle	$k_{s3}\sim k_{s6}$	kN/m	-	-	500
Spring constant of suspension of rear axle	$k_{s7}\sim k_{s12}$	kN/m	22	500	500
Damping coefficient of suspension of front axle	$c_{s1},c_{s2}$	kN·s/m	1.5	10	10
Damping coefficient of suspension of middle axle	$c_{s3}\sim c_{s6}$	kN·s/m	-	-	15
Damping coefficient of suspension of rear axle	$c_{s7}\sim c_{s12}$	kN·s/m	1.5	15	15
Spring constant of tire of front axle	$k_{w1},k_{w2}$	kN/m	192	500	600
Spring constant of tire of middle axle	$k_{w3}\sim k_{w6}$	kN/m	-	-	1200
Spring constant of tire of rear axle	$k_{w7}\sim k_{w12}$	kN/m	192	1000	1200
Damping coefficient of tire of front axle	$c_{w1},c_{w2}$	kN·s/m	0	1.5	2
Damping coefficient of tire of middle axle	$c_{w3}\sim c_{w6}$	kN·s/m	-	-	4
Damping coefficient of tire of rear axle	$c_{w7}\sim c_{w12}$	kN·s/m	0	3	4

**Table 7:** Fatigue life estimation under different road roughness.

Fatigue Life (year)	Suspender A	Suspender B	Suspender C	Suspender D	Suspender E	Suspender F
Range_A	7006	6951	4701	6113	1468	1734
Range_E	186	289	105	139	598	792

**Table 8:** Fatigue life estimation under different road roughness considering corrosion effects.

Fatigue Life (year)	Suspender A	Suspender B	Suspender C	Suspender D	Suspender E	Suspender F
Range_A	5.0	5.0	4.8	4.7	8.8	8.6
Range_E	1.7	1.9	1.6	1.7	2.9	2.9

## Data Availability

The WIM data used in this research cannot be shared because of the confidentiality agreement from the bridge maintenance company.

## Nomenclature

$M_{v},\;C_{v},\;K_{v}$	mass matrix, damping matrix and stiffness matrix of vehicle
$F_{v}$	column vector of external load of vehicle
$z_{vj},\alpha_{vj},\beta_{vj}$	vertical displacement, pitching rotation, and rolling rotation of vehicle body part j
$I_{vj},\;J_{vj}$	pitching inertia moment and rolling inertia moment of vehicle body part j
$z_{i}$	vertical displacement of wheel i
$k_{wi},c_{wi}$	stiffness coefficient and damping coefficient of wheel i
$z_{gi},\;{\dot{z}}_{gi}$	displacement excitation and velocity excitation of wheel i
$r\left(x_{i}\right)$	road roughness of wheel i in location x
$z_{xi}$	instantaneous displacement of bridge at the location of wheel i
$k_{si},c_{si}$	stiffness coefficient and damping coefficient of suspension system i
$M_{b},C_{b},\;K_{b}$	total mass matrix, damping matrix, and stiffness matrix of bridge structure
$F_{b}$	column vector of external force of bridge structure
$z_{b}$	column vector of bridge node
$F_{i}$	road instantaneous force from wheel i
$W_{i}$	static axle load of wheel i
$S\left(\omega_{k}\right)$	power spectral density function of road roughness
${\dot{r}}_{v}\left(x\right)$	velocity term of road roughness
${\dot{r}}_{x}\left(x\right)$	change rate of road roughness
D′	damage degree
S	stress range
car_1.5	representative vehicle type: 1.5 tons car
truck_15	representative vehicle type: 15 tons truck
trailer_50	representative vehicle type: 50 tons trailer
DOF	degree of freedom

## References

[1] Fu Z., Ji B., Yang M., Sun H. (2015). Cable replacement method for cable-stayed bridges based on sensitivity analysis. J. Perform. Constr. Facil..

[2] Bedon C., Fasan M. (2019). Reliability of field experiments, analytical methods and pedestrian’s perception scales for the vibration serviceability assessment of an in-service glass walkway. Appl. Sci..

[3] Yang S.C. (2018). Research on the Corrosion-Fatigue Problems and Service Reliability of the Bridge Cables and Hangers.

[4] Liu Z., Guo T., Hebdon M.H., Zhang Z. (2018). Corrosion fatigue analysis and reliability assessment of short suspenders in suspension and arch bridges. J. Perform. Constr. Facil..

[5] Cui C., Ma R., Chen A., Pan Z., Tian H. (2019). Experimental study and 3D cellular automata simulation of corrosion pits on Q345 steel surface under salt-spray environment. Corros. Sci..

[6] Bedon C., Dilena M., Morassi A. (2016). Ambient vibration testing and structural identification of a cable-stayed bridge. Meccanica.

[7] Kawatani M., Komatsu S. (1988). Nonstationary Random Response of Highway Bridges under a Series of Moving Vehicles. Struct. Eng. Earthq. Eng. JSCE.

[8] Zhang W., Cai C. (2012). Fatigue reliability assessment for existing bridges considering vehicle speed and road surface conditions. J. Bridge Eng..

[9] Zhang W., Cai C., Pan F. (2013). Nonlinear fatigue damage assessment of existing bridges considering progressively deteriorated road conditions. Eng. Struct..

[10] Wang T.-L., Liu C., Huang D., Shahawy M. (2005). Truck loading and fatigue damage analysis for girder bridges based on weigh-in-motion data. J. Bridge Eng..

[11] Wang W., Deng L., Shao X.D. (2016). Fatigue design of steel bridges considering the effect of dynamic vehicle loading and overloaded trucks. J. Bridge Eng..

[12] Alencar G., de Jesus A.M.P., Calcada R.A.B., da Silva J.G.S. (2018). Fatigue life evaluation of a composite steel-concrete roadway bridge through the hot-spot stress method considering progressive pavement deterioration. Eng. Struct..

[13] Li Y., Ma X., Zhang W., Wu Z. (2018). Updating time-variant dimension for complex traffic flows in analysis of vehicle–bridge dynamic interaction. J. Aerosp. Eng..

[14] Liu Z., Guo T., Huang L., Pan Z. (2017). Fatigue life evaluation on short suspenders of long-span suspension bridge with central clamps. J. Bridge Eng..

[15] Henchi K., Fafard M., Talbot M., Dhatt G. (1998). An efficient algorithm for dynamic analysis of bridges under moving vehicles using a coupled modal and physical components approach. J. Sound Vib..

[16] Au F.T.K., Cheng Y.S., Cheung Y.K. (2001). Effects of random road surface roughness and long-term deflection of prestressed concrete girder and cable-stayed bridges on impact due to moving vehicles. Comput. Struct..

[17] Kim C.W., Kawatani M., Kim K.B. (2005). Three-dimensional dynamic analysis for bridge-vehicle interaction with roadway roughness. Comput. Struct..

[18] Lin L. (2014). Evaluation of Comfort Level Based on Vehicle-Bridge Coupling Analysis Research for Steel Arch Bridge with Special-Shape.

[19] Hwang E.S., Nowak A.S. (1991). Simulation of dynamic load for bridges. J. Struct. Eng..

[20] Yang J.R., Li J.Z. (2010). Local dynamic response in deck slab of a concrete box girder bridge. J. Sichuan Univ. (Eng. Sci. Ed.).

[21] Li X.N. (2014). Theoretical Research on Bridge Weigh-in-Motion System Based on Monitored Strain.

[22] Szurgott P., Wekezer J., Kwasniewski L., Siervogel J., Ansley M. (2010). Experimental assessment of dynamic responses induced in concrete bridges by permit vehicles. J. Bridge Eng..

[23] Sarkar S., Gupta S., Rychlik I. (2011). Wiener chaos expansions for estimating rain-flow fatigue damage in randomly vibrating structures with uncertain parameters. Probabilistic Eng. Mech..

[24] Huang W. (2017). The frequency domain estimate of fatigue damage of combined load effects based on the rain-flow counting. Mar. Struct..

[25] Shelton S.M., Swanger W.H. (1935). Fatigue properties of steel wire. J. Res. Natl. Bur. Stand..

[26] Zhang J.N. (2016). Study on Corrosion and Fatigue Properties of High-Strength Galvanized Steel Wire Used for Cable of Bridge.

[27] Zheng X.L. (2018). Research on the Fatigue Performance of Corroded Steel Wire and Evaluation Method of Fatigue Reliability for Bridge Cables.

